# Thoracotomy versus VATS: is there an optimal approach to treating pneumothorax?

**DOI:** 10.1308/003588413X13511609956138

**Published:** 2013-01

**Authors:** V Joshi, B Kirmani, J Zacharias

**Affiliations:** Blackpool Teaching Hospitals NHS Foundation TrustUK

**Keywords:** Pleural disease, Pleurectomy, Thoracic surgery, Video-assisted thoracoscopic surgery

## Abstract

**Introduction:**

The 2010 British Thoracic Society guidelines recommend that a weighted decision be made by clinicians with regard to surgical intervention for pneumothorax as the video assisted thoracoscopic surgery (VATS) approach is better tolerated by patients but carries a higher rate of recurrence (5% vs 1%).

**Methods:**

Overall, 163 patients underwent surgical intervention for pneumothorax at our institution and data were collected prospectively for almost 7 years. Of these, 86 patients underwent VATS under a single surgeon with extensive VATS experience to compensate for the associated learning curve while 79 patients underwent an open procedure.

**Results:**

There was no statistically significant difference in the recurrence rate between the open and the VATS group (1% vs 3.5%, *p*=1.0). The VATS group was superior to the open group in terms of reduced postoperative bleeding (7.5% vs 0%, *p*=0.01), reduced number of intensive care unit admissions (16% vs 0%, *p*<0.01) and a reduced adjusted length of stay (3 vs 5.5 days, *p*<0.01).

**Conclusions:**

A comparable recurrence rate is attainable with a VATS approach once the learning curve is surpassed and a reduction in morbidity is an additional merit.

The overall incidence of pneumothorax was found to be 24 and 9.8 per 100,000 for men and women respectively.^1^ The incidence of primary spontaneous pneumothorax (PSP) has been reported at 7.7 and 1.3 per 100,000 for men and women respectively.^2^ The definitive method of treatment for PSP is a matter of debate. British Thoracic Society guidelines on the management of pneumothorax were released in 2010, recommending that a weighted decision be made by clinicians as the video-assisted thoracoscopic surgery (VATS) approach is better tolerated by patients but carries a higher rate of recurrence (5% vs 1% for open surgery).^3^

Like any minimally invasive technique, VATS procedures have an associated learning curve. Once the learning curve is surpassed, complications are less likely to be prevalent and optimum results can be observed.^4–8^ In this study, we sought to compare traditional open with VATS approaches at a single centre for treating pneumothorax. The primary outcome measure was recurrence of pneumothorax with secondary outcome measures including in-hospital procedural failure, bleeding, intensive care unit (ICU) admission and length of stay.

## Methods

Patient data were collected prospectively and entered into a thoracic surgical database between 2004 and mid-2011. A total of 163 patients were identified as having undergone surgical intervention for pneumothorax. All patients were included who underwent an open procedure at our institution (*n*=79) as well as all patients of a surgeon who exclusively uses a VATS approach for treating pneumothorax (*n*=86). This was to minimise procedural heterogeneity or the effects of a ‘learning curve’ by limiting the VATS cohort to a single surgeon with sufficient experience in minimally invasive techniques.

**Table 1:** Patient characteristics for study population

Characteristic	Open procedure (*n*=79)	VATS (*n*=86)	*p*-value
Male	59 (75%)	60 (70%)	0.49
Female	20 (25%)	26 (30%)	0.49
Median age (IQR)	34 (22–48)	33 (22–57)	0.44
Pleurectomy	74 (94%)	72 (84%)	1.0
Talc pleurodesis	5 (6%)	14 (16%)	1.0

VATS = video-assisted thoracoscopic surgery;

IQR = interquartile range

At our institution, the standard VATS approach to a patient with pneumothorax includes an apical to mid-level pleurectomy and basal pleural abrasion. Our open approach is surgeon dependent. It usually constitutes a bullectomy and either a pleurectomy or talc pleurodesis via a mini-posterolateral thoracotomy. Talc insufflation was used primarily for complicated cases or secondary pneumothorax in either group. There were no conversions in the VATS group to an open procedure.

**Table 2:** Outcome measures

Characteristic	Openprocedure (*n*=79)	VATS (*n*=86)	*p*-value
Bleeding	6 (8%)	0 (0%)	**0.01**
Recurrence	1 (1%)	3 (3%)	1.0
Air leak	14 (18%)	8 (9%)	0.16
Talc on ward	2 (23%)	1 (1%)	0.61
ICU admission	13 (16%)	0 (0%)	**<0.01**

VATS = video assisted thoracoscopic surgery;

ICU = intensive care unit

Patients were divided into two subgroups depending on which type of intervention they received: either bullectomy and pleurectomy alone for PSP (74 and 72 for open and VATS groups respectively) or bullectomy and talc pleurodesis for secondary pneumothorax (5 and 14 for open and VATS groups respectively). No other statistically significant differences existed between the open and VATS groups in terms of general patient demographics. The patient demographics are shown in Table 1.

The adjusted length of stay was calculated by excluding patients who stayed longer than 14 days (8 and 9 patients for open and VATS groups respectively). We believe this to be a more accurate reflection of length of stay as a result of the surgical approach. A prolonged hospital stay was usually a result of a complicated underlying disease process or social reasons.

A case was considered to be a recurrence if the patient was readmitted following discharge with a subsequent pneumothorax on a previously operated side. Recurrent pneumothoraces were discovered following investigation for recurrent symptoms or during routine six-week followup. At our centre, patients are referred back to our service if they develop a recurrent pneumothorax at any time following operative intervention. No limitation was therefore placed on the follow-up duration.

Statistical tests were performed with Prism^®^ (GraphPad Software, La Jolla, CA, US). Analysis of continuous data was performed using unpaired t-tests and categorical data using Fisher’s exact test. A *p*-value of <0.05 was considered statistically significant.

## Results

Of the 79 patients who underwent open surgical intervention, only 1 (1%) needed a ‘re-do’ procedure. Of the 86 patients who underwent VATS intervention, 3 (3%) needed a re-do procedure. There was no statistically significant difference in recurrence rates between the two groups (*p*=1.0) (Table 2). Two of the three patients needing repeat intervention had a history of substance abuse, which may have been a contributing factor towards recurrence in those cases. Video-assisted talc pleurodesis was performed in these patients and there were no further recurrences.

**Figure 1:**
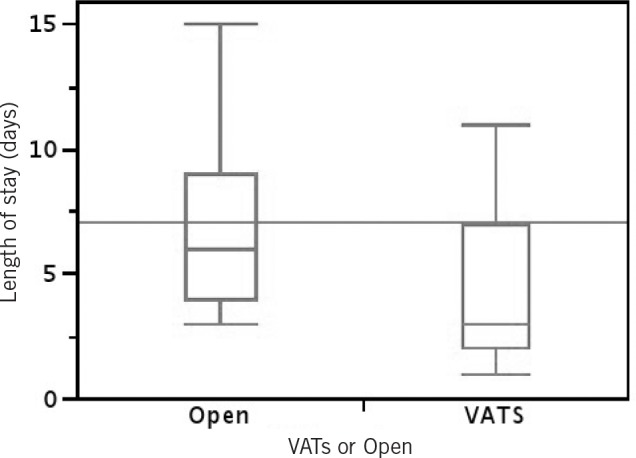
Box-and-whisker plot comparing the adjusted length of stay for both the video-assisted thoracoscopic surgery (VATS) and open group. Interquartile range depicted in box with median represented by horizontal line. Sample range depicted above and below.

There were no statistically significant differences between the open and VATS groups with regard to persistent air leak greater than seven days (18% vs 9% respectively, *p*=0.16) or the need for additional talc pleurodesis during the same admission (3% vs 1% respectively, *p*=0.61). Patients who underwent open surgical intervention were more likely to need re-exploration for excessive bleeding compared with the VATS group (8% vs 0%, *p*=0.01) and were more likely to need admission to ICU for short-term monitoring (16% vs 0%, *p*<0.01). There was no statistically significant difference between the open and VATS groups in terms of median length of stay (6 vs 4 days respectively, *p*=0.46). However, when patients who remained in hospital longer than two weeks were excluded, the median adjusted length of stay was found to be shorter for the VATS group than the open group (3 vs 5.5 days, *p*<0.01) (Fig 1).

There were no major complications in terms of patients requiring organ or system support, or mortality. All patients needing re-do surgery had their second operation with a similar approach to their first. However, talc was used for pleurodesis irrespective of the primary operation. There were no further recurrences in either group.

## Discussion

Guidelines on the management of pneumothorax released have highlighted a recurrence rate of approximately 5% with a VATS approach.^3^ The meta-analysis by Barker *et al* demonstrated a fourfold increase in the recurrence rates in patients undergoing VATS.^9^ It is the authors’ belief that outcomes from older studies may be affected negatively by the learning curve associated with achieving better results with VATS. Both Bertrand *et al* and Mouroux *et al* highlighted this in their studies in 1996 as a possible limitation to attaining similar results to thoracotomy.^7,10^ This would also explain why the retrospective study by Sawada *et al* looking back at a similar time period showed high recurrence rates with a VATS approach when compared with current standards.^11^ Waller emphasised the importance of the learning curve in VATS procedures for pneumothorax.^8^ He noted an inversely proportional relationship between recurrence rates and experience in his study population over a seven-year period.

In our study, the cohort of patients undergoing a VATS approach had their operation performed by a single surgeon with a routine VATS practice. We were able to demonstrate a recurrence rate of less than 5% over almost seven years’ practice with no limitation on the length of follow-up. This was, however, not statistically different to the recurrence rate in the open group.

Previous work evaluating the benefits of a VATS approach has shown it to result in fewer complications postoperatively such as reduced bleeding, pain and pulmonary dysfunction.^3,12–15^ Additionally, the cosmetic advantages of minimally invasive techniques make them more appealing to younger patients. Our data have shown a statistically significant reduction in postoperative bleeding necessitating re-exploration as well as a reduction in the need for ICU monitoring if VATS is used. Our data also suggest patients undergoing VATS procedures are less likely to have a prolonged postoperative air leak for more than seven days compared with open procedures. Although this was not statistically significant in our study, Crisci and Coloni have had similar results.^16^ We observed a shorter median length of stay with the VATS approach, which is similar to findings from other studies. In the current era of health economics, a reduced length of stay equates to greater efficiency.^12,16,17^

At our institution, an apical to midlevel pleurectomy and basal pleural abrasion are the mainstay of the VATS approach. This method has been described previously and may optimise results.^18,19^ VATS is associated with a less complicated postoperative course. Nevertheless, the question of the reason for the higher recurrence rate still remains. By limiting the minimally invasive cohort of patients to those operated on by an experienced VATS practitioner, it was hoped that the effects of the VATS learning curve on recurrence rates could be eliminated, producing outcomes approaching that of the standard open approach. Although there was no statistically significant difference, our data indicated a trend towards higher recurrence rates in the VATS group, even in the context of reduced postoperative complications.

The reasons for higher recurrence rates in minimally invasive procedures for pneumothoraces have not yet been established. One hypothesis is that the open approach allows for greater visualisation and therefore a more extensive pleurectomy, which correlates directly with a lower recurrence rate.^3^ Additionally, a minimally invasive approach results in reduced levels of both inflammatory and vasoactive mediators such as C-reactive protein, prostacyclin and thromboxane A2 when compared with thoracotomy. This may not be favourable when the desired pleurodesis is a sequelae of the inflammatory response.^19,20^

There is a recognised link between tobacco smoking and the development of PSP, and a negative effect has been observed in regard to the recurrence rate following surgical intervention.^21,22^ A less defined link exists between the development of pneumothorax and illicit drug use. There have been several cases suggesting that an association may exist.^23,24^ In our study population, two of the three patients in the VATS group with recurrent pneumothorax following surgery had a history of illicit drug use. The use of cannabis is associated with bullous changes in the lung and the repetitive use of cocaine can induce pulmonary barotrauma, both of which can result in pneumothorax.^25,26^ It is our standard practice now to perform talc pleurodesis in all patients with a history of illicit drug use presenting with PSP during primary surgery as this may improve long-term recurrence free outcome.^27^

Our study population was limited to patients of only a single surgeon routinely performing VATS procedures at our institution. Despite the limitations to our sample size, a trend was observed for patients with a history of substance abuse towards developing recurrent pneumothorax. A large registry comprising patient data from multiple thoracic surgeons with routine VATS practice may help identify other possible groups of patients at a higher risk for recurrence. Analysis of pooled data may allow us to discover how we can improve the long-term results of the VATS approach while maintaining the observed short-term and cosmetic advantages, which would be the ideal therapeutic option.

## Conclusions

Although a comparable recurrence rate is achievable with a VATS approach, further studies with larger sample sizes are needed to demonstrate statistically valid results. VATS is associated with less bleeding, a reduction in ICU admission and a shorter length of stay.
